# Gene expression of fibrinolytic markers in coronary thrombi

**DOI:** 10.1186/s12959-022-00383-1

**Published:** 2022-04-29

**Authors:** Jostein Nordeng, Svein Solheim, Sissel Åkra, Hossein Schandiz, Pavel Hoffmann, Borghild Roald, Bjørn Bendz, Harald Arnesen, Ragnhild Helseth, Ingebjørg Seljeflot

**Affiliations:** 1 Center for Clinical Heart Research, Oslo University Hospital Ullevål, Kirkeveien 166, Pb 4950 Nydalen, N-0424 Oslo, Norway; 2 Department of Cardiology, Oslo University Hospital Ullevål, Kirkeveien 166, Pb 4950 Nydalen, N-0424 Oslo, Norway; 3 Faculty of Medicine, University of Oslo, Klaus Torgårds vei 3, Pb 1078 Blindern, 0316 Oslo, Norway; 4 Department of Pathology, Akershus University Hospital, Sykehusveien 25, Pb1000, 1478 Lørenskog, Norway; 5 Section for Interventional Cardiology, Department of Cardiology, Oslo University Hospital Ullevål, Kirkeveien 166, Pb 4950 Nydalen, N-0424 Oslo, Norway; 6 Department of Pathology, Oslo University Hospital Ullevål, Kirkeveien 166, Pb 4950 Nydalen, N-0424 Oslo, Norway; 7 Department of Cardiology, Oslo University Hospital Rikshospitalet, Sognsvannsveien 20, Pb 4950 Nydalen, N-0424 Oslo, Norway

**Keywords:** Coronary thrombi, Fibrinolytic system, ST-elevation infarction, Genetic expression

## Abstract

**Background:**

The fibrinolytic system plays an important role in coronary artery atherothrombosis, and especially circulating plasminogen-activator inhibitor (PAI) type 1 (PAI-1) associates with increased mortality, infarct size and heart failure in patients with myocardial infarction (MI). In a cross-sectional study, we aimed to study whether genes encoding tissue plasminogen activator (tPA), urinary-type plasminogen activator (uPA), PAI-1 and PAI-2 are expressed in coronary thrombi from acute ST-elevation MI (STEMI) patients. Any relations to myocardial injury measured by peak troponin T, time from symptom onset to Percutaneous Coronary Intervention (PCI), and to different cell types present in the thrombi were also explored.

**Methods:**

Intracoronary thrombi were aspirated from 33 STEMI patients treated with primary PCI. The thrombi were snap-frozen for gene expression analyses, relatively quantified by RT PCR. Peripheral blood samples were drawn. Correlations were performed by Spearmans rho.

**Results:**

The genes were present in 74–94% of the thrombi. Median peak troponin T was 3434 μ/L and median ischemic time 152 min. There were no significant correlations between the measured genes and troponin T, or ischemic time. Genes encoding tPA, u-PA, PAI-1 and PAI-2 all correlated significantly to the presence of monocytes/macrophages (CD68) in the thrombi (*p* = 0.028, *p* < 0.001, *p* = 0.003, *p* < 0.001). PAI-1 and PAI-2 also correlated to endothelial cells (CD31) (*p* = 0.002, *p* = 0.016). uPA associated with neutrophil granulocytes (CD 66b) (*p* = 0.019).

**Conclusion:**

Genes encoding tPA, uPA, PAI-1 and PAI-2 were highly expressed in human coronary thrombi from STEMI patients, indicating fibrinolytic regulators playing active roles in the thrombi, although not related to myocardial injury. All markers related to the presence of monocytes/macrophages, indicating connection to local inflammatory cells.

**Trial registration:**

The study is registered at clinicaltrials.gov with identification number NCT02746822.

**Supplementary Information:**

The online version contains supplementary material available at 10.1186/s12959-022-00383-1.

## Background

Activation of the coagulation system results in formation of cross-linked fibrin, and is balanced by the fibrinolytic system. Both tissue Plasminogen Activator (tPA) and urinary-type Plasminogen Activator (uPA) facilitate the conversion of plasminogen to plasmin, which causes lysis of cross-linked fibrin. tPA is located mainly in endothelial cells and in the circulation on the surface of fibrin, accelerating the converting process and considered important for fibrinolysis. uPA exerts its effects mainly on cellular surfaces, and is more related to the effects of plasmin on matrix remodeling in tissue [1]. Plasminogen-activator inhibitor type 1 (PAI-1) is the main fibrinolytic inhibitor, inhibiting both tPA and uPA in a fast reaction [2], whereas type 2 (PAI-2), localized predominantly intracellularly, is a slower inhibitor, with higher affinity for uPA [3]. PAI-2 is normally undetectable in the circulation in humans, in contrast to PAI-1 which also acts as an acute phase protein [4].

Knowledge of the fibrinolytic system has for years been utilized in treatment of thromboembolic disorders like acute myocardial infarction, stroke or pulmonary embolism, by intravenous administration of recombinant tPA, or other plasminogen activators, dissolving the harming thrombus [5]. Beyond the thromboembolic diseases, fibrinolytic activators and their regulators have also been associated with coronary artery atherothrombosis [6]. Plasma levels of tPA and PAI-1 have been found to predict myocardial infarction, and high levels associate with poor prognosis in populations with high prevalence of coronary artery disease [7], and in patients with chronic coronary syndrome [8]. PAI-1 has also been found to associate with established risk factors for atherosclerotic disease, such as insulin resistance/type 2 diabetes [9] and cholesterol levels [10]. PAI-1 activity has moreover been related to increased mortality, infarct size and heart failure in patients with STEMI [11, 12]. Animal models also indicate that PAI-1 can mediate cardiac fibrosis [13] and is involved in remodeling after myocardial infarction [14], but also that it might confer protection against cardiac rupture [15].

To better understand the role of these specific fibrinolytic markers in an acute myocardial infarction, we aimed to investigate the genetic expression, presence and localization of tPA, uPA, PAI-1 and PAI-2 in coronary thrombi from STEMI patients. We hypothesized all markers to be present and actively regulated. A set of cell markers, indicating what cell types related to the fibrinolytic markers, were also assessed. We furthermore explored associations with degree of myocardial injury and time from symptoms to PCI. Gene expression levels in circulating leukocytes were also measured, as well as PAI-1 levels in circulation.

## Methods

### Study design and population

Details of the Thrombus Aspiration in ST-elevation myocardial Infarction (TASTI) study has been described previously [16]. Thirty-three patients admitted to Oslo University Hospital Ullevål in the time period of August 2015 to January 2019 with ST-elevation myocardial infarction (STEMI) treated with primary PCI and thrombus aspiration were consecutively included. All participants signed a written informed consent. The study was approved by the Regional Committee of Medical Research Ethics in South-Eastern Norway with approval reference number 2015/169, and it is registered at clinicaltrials.gov with identification number NCT02746822. The study was conducted according to the Declaration of Helsinki.

After retrieval with standard aspiration catheter, coronary thrombi were washed with saline and split in two. One part was snap-frozen in RNA-later solution (Qiagen, Hilden, Germany) and kept frozen at − 80 °C for later isolation of RNA and gene expression analyses. The other part was stored in 10% buffered formalin, chemically processed and paraffin embedded for later histology and immunohistochemistry analyses. Blood samples were drawn at the end of the PCI-procedure and the morning after. Blood without additives was collected for serum preparation, i.e. centrifuged at 2500 x g for 10 min and stored at − 80 °C until analysis. Tubes containing 0.129 M trisodium citrate (3.8%) in dilution 1:10, were collected and kept on ice until preparation of citrated plasma by centrifugation at 4 °C for 20 min within 30 min, and stored at − 80 °C until analysis. PAXgene tubes (PreAnalytix, Hombrechtikon, Switzerland) were collected and kept at room temperature at least 2 h, and kept frozen at − 80 °C until RNA extraction. Cardiac troponin T values (cTnT) were measured by commercial electrochemiluminescence immunoassay (third generation cTnT, Elecys 2010, Roche, Mannheim, Germany) at the hospital central laboratory. The inter-assay coefficient of variability was 7%. Demographic variables were recorded in case record forms.

### Laboratory analyses

#### Gene expression analyses

High Pure RNA Tissue Kit, with addition of Proteinase K Solution (Roche Diagnostics GmbH, Mannheim, Germany), was used for isolating RNA from the snap-frozen part of the thrombi, after they were stabilized by lysing buffer and homogenized by use of termomixer (Termomixer Eppendorf, Eppendorf AG, Hamburg, Germany) and Stainless steel grinding balls (Qiagen GmbH, Hilden, Germany). PAXgene® Blood RNA Kit (PreAnalytix, Qiagen, GmBH), with an extra cleaning step (RNeasy®MinElute® Cleanup Kit, Qiagen), was used to isolate RNA from the PAXgene tubes. Quality and quantity of RNA (ng/μL) were evaluated by use of the NanoDrop™ 1000 Spectrophotometer (Thermo Scientific, Wilmington, Delaware, USA). The purity, assessed as the 260/280 nm absorbance ratio, was mean 1.6. Mean quantity was 27.6 ng/μL.

cDNA was synthesised from equal amount of RNA with qScript™ cDNA superMix (Quanta Biosciences, Gaithersburg, Maryland, USA). Real-time PCR was performed on a ViiA™ 7 instrument (Applied Biosystems, Foster City, CA, USA) using TaqMan® Universal PCR Master Mix (P/N 4324018) and the following TaqMan® assays: TPA (Hs00263492_m1), UPA (Hs01547054_m1), PAI-1 (Hs01126606_m1), PAI-2 (Hs01010736_m1), CD3 (Hs00609515_m1), CD68 (Hs00154355_m1), CD31(Hs00169777_m1), ACTA2/α-SMA (Hs_00426835_g1) and CD 61 (Hs01001469_m1). β2-microglobulin (HS99999907_m1) (Applied Biosystems) was used as endogenous control. Relative quantification (RQ) by the Delta Delta C(T) Method (DDCT) [17] was used for determining mRNA levels. A relative quantification (RQ) value of 1.0 indicates that the gene analyzed was equally expressed as the reference sample.

#### Circulating levels

Citrated blood was used for determination of PAI-1 activity. A commercially available chromogenic assay (Spectrolyse PAI-1, BioMedica Diagnostics, Windsor, NS, Canada) was used. Coefficient of variation in our laboratory was 8.7%.

#### Histology and immunohistochemistry-analyses

The paraffin embedded thrombi were cut in serial sections at 3.5 μm. Every 5th section was stained with hematoxylin and eosin (HE). Morphological staging into different thrombus age groups was done from the HE sections. This was done according to the criteria listed in Supplementary Table 1, Additional File 1. These are based on commonly used (Carol [18] and Rittersma [19] among others) previous criteria. In our modified classification, we added the presence of increasing number of monocytes as a characteristic of stage 2, and also introduced stage 1+, with early signs of disintegration of granulocytes but no presence of monocytes. Dako Autostainer System (Aglient Technologies, Santa Clara, California, USA), an automated immunostaining system based on the ABC avidin-biotin-peroxidase method, was used for immunohistochemistry on serial sections of the thrombi. Optimal antigen retrieval, antibody concentrations and incubation times were pre-tested with positive and negative controls for the antibodies tPA, uPA, PAI-1 and PAI-2 (Supplementary Table 2, Additional File 2). Immunoreactivity was recorded in relation to cell type and localization, and thrombi were classified as either immunohistochemical immune-enzyme negative (IH negative) or positive (IH positive) for the different antibodies.

Two pathologists performed histopathological judgements individually. Inter-observer agreement was tested for the morphological classification and the immunohistochemical scoring of the antibody signals, and was substantial with Kappa scores [20] of 0.82 to 0.86.

### Statistical analyses

Data are presented as mean ± SD, median (25th, 75th percentile) or numbers (%) as appropriate. Most variables were skewed distributed, hence non-parametric tests were used. Spearman rho was used for correlation analyses. Mann-Whitney U-test and Kruskal-Wallis tests were used when comparing groups, and Wilcoxon signed-rank test for pairwise comparisons. Missing values were excluded from the analyzes. *P*-values ≤0.05 were considered statistically significant. The analyses were performed using STATA IC/15.1.

## Results

### Baseline characteristics

Median age of the 33 patients was 58 years. 91% were male, 49% were current smokers and 12% had diabetes. Median peak troponin T was 3434 μg/L, whereas median time from start of symptoms to PCI was 152 min. The culprit lesion was located in the left anterior descending artery (LAD) in 49% of the patients. The baseline characteristics are displayed in Table 1.

**Table 1:** Baseline characteristics

Female sex	3 (9%)
Age (yrs)	58.0 (54.0, 68.0)
Current smokers	16 (49%)
Previous smokers	11 (33%)
BMI (kg/m^2^)	27.7 (23.4, 28.6)
Hypertension	11 (33%)
T2DM	4 (12%)
Previous coronary disease	1 (3%)
Medication before index MI:	
ASA	6 (18%)
Clopidogrel/Prasugrel/Ticagrelor	2 (6%)
Warfarin	1 (3%)
NOAC	2 (6%)
Betablocker	4 (12%)
ACE-I	0 (0%)
AT-II-blocker	5 (15%)
Statins	6 (18%)
Diuretics	4 (12%)
Aldosterone antagonists	0 (0%)
Systolic BP (mmHg)	126.0 (109.0, 144.0)
Diastolic BP (mmHg)	80.0 (70.0, 99.5)
HR (beats/min)	70.0 (65.0, 90)
Ischemic time (min)	152 (122, 343)
CRP (mg/L)	2.71 (1.00, 5.57)
Troponin T after PCI (ng/L)	354 (123, 744)
Troponin T peak (ng/L)	3434 (1250, 6967)
Culpruit lesion:	
LAD	16 (49%)
CX	6 (18%)
RCA	11 (33%)
Retrograde flow	12 (36%)
Three-vessel disease	6 (18%)
In-stent thrombus	1 (3%)

Baseline characteristics of the population (*n* = 33). Values are given as absolute numbers (%) or medians (25, 75 percentiles). BMI = Body Mass Index. T2DM = Type II Diabetes Mellitus. ASA = Acetysalisylic acid. NOAC = Novel Oral AntiCoagulant. ACE-I = Angiotensin Converting Enzyme Inhibitor. AT-II-blocker = Angiotensin receptor II -blocker. BP = Blood Pressure. HR = Heart Rate. CRP = C-Reactive Protein. PCI = Percutaneous Coronary Intervention. LAD = Left Anterior Descending Artery. CX = Circumflex artery. RCA = Right Coronary Artery.

### Gene expression analyses and circulating levels

Of the 33 aspirated thrombi, 31 were successfully analyzed for gene expression. Analyzes of gene expression in circulating leukocytes could be done for all patients (*n* = 33) at time of PCI for uPA, PAI-1 and PAI-2, whereas five were missing for tPA (*n* = 28). In samples collected the next day four were missing for uPA and PAI-1 (*n* = 29), five were missing for PAI-2 (*n* = 28), and seven were missing for tPA (*n* = 26). Missing values were due to non-successful analyses. PAI-1 in circulation was successfully analyzed for all patients at time of PCI, whereas the next day, one was missing (*n* = 32), due to lack of blood sample.

**Fig. 1:**
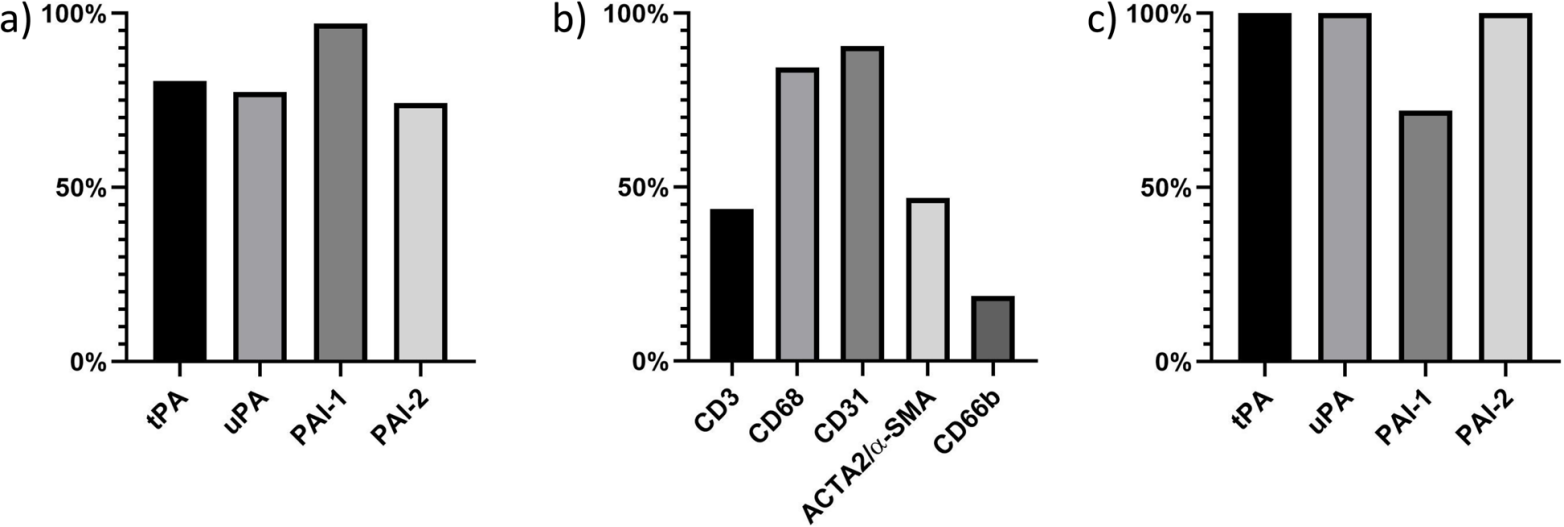
Genes expressed and immunohistochemistry staining in thrombi. Panel **a** shows percentage of thrombi with the fibrinolysis-related genes expressed (*n* = 31). Panel **b** shows percentage of thrombi with the cell-marker genes expressed (*n* = 32). Panel **c** shows percentage of thrombi with immunohistochemistry staining of the fibrinolytic markers (*n* = 26)

Genes coding for the four markers were present in 74–97% of the successfully analyzed thrombi (Fig. 1a). Genes encoding PAI-1 were found in 97%, tPA in 81%, uPA in 77% and PAI-2 in 74%. Of the cell-marker genes, CD31 (representing endothelial cells) was found in 91% (Fig. 1b), CD68 (monocytes/macrophages) in 84%, ACTA2/α-SMA (smooth muscle cells) in 47%, CD3 (T-cells) in 44% and CD66b (neutrophil granulocytes) in 19%.

In circulating leukocytes, all markers were expressed in 100% of the available samples both at the time of PCI and the next day. Levels of gene expression (RQs) in the aspirated thrombi and in circulating leukocytes at the time of aspiration and the next morning (Day 1), as well as plasma levels at the same time points, are shown in Supplementary Table 3, Additional File 3.

We found no significant correlations between genetic expression of PAI-1 in thrombi and levels of PAI-1 in circulation at any timepoint (Supplementary Table 4, Additional File 4). There was an inverse significant correlation between gene expression in circulating leukocytes at the time of PCI and circulating levels of PAI-1 at Day 1 (*p* = 0.024). No significant correlations were found between gene expression of the markers in the thrombi and expression of corresponding markers in circulating leukocytes at either time points (data not shown).

#### Associations with myocardial injury (Table 2)

**Fig. 2:**
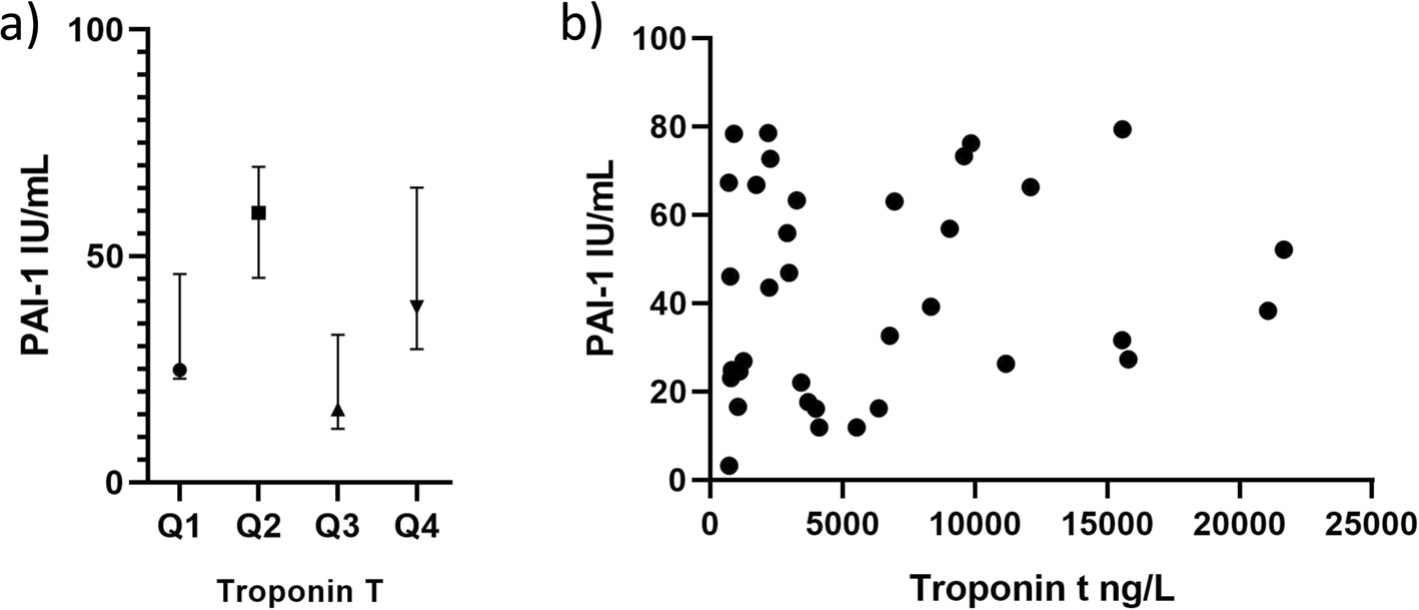
PAI-1 and troponin T. PAI-1 in circulation at Day 1, in panel **a** related to quartiles (Qs) of troponin T (Kruskal Wallis rank test), in panel **b** as a scatterplot of peak Troponin T

There were no significant correlations between genes expressed in thrombi and peak troponin T. The same was found for genes in circulating leukocytes and circulating PAI-1, both at time of PCI and at Day 1. Also after dichotomizing data at median level of troponin T (3434 ng/L), or divided into quartiles (Qs)1–3 vs Q4, no significant differences were found, other than a cross quartile significant difference (Kruskal Wallis rank test) in circulating PAI-1 (*p* = 0.020), but as shown in Fig. 2 this was due to wide variation.

**Table 2:** Relation to peak troponin T

At time of PCI						
	Genes in thrombus		Genes in circ.leuk.		Circulating marker	
	Rho	*p*	Rho	*p*	Rho	*p*
tPA	−0.142	0.498	−0.222	0.256	–	–
uPA	0.137	0.522	0.151	0.402	–	–
PAI-1	−0.156	0.411	− 0.109	0.547	− 0.205	0.253
PAI-2	0.342	0.110	0.078	0.667	–	–
At Day 1						
	Genes in thrombus		Genes in circ.leuk.		Circulating marker	
	Rho	*p*	Rho	*p*	Rho	*p*
tPA	–	–	−0.113	0.582	–	–
uPA	–	–	0.090	0.641	–	–
PAI-1	–	–	−0.041	0.831	0.050	0.785
PAI-2	–	–	0.247	0.206	–	–

Coefficients of correlation (Spearmans rho) between peak troponin T and the markers measured at time of PCI and at Day 1. *p* ≤ 0.05 bolded as sign of statistical significance

#### Associations with time from symptom onset to PCI

**Fig. 3:**
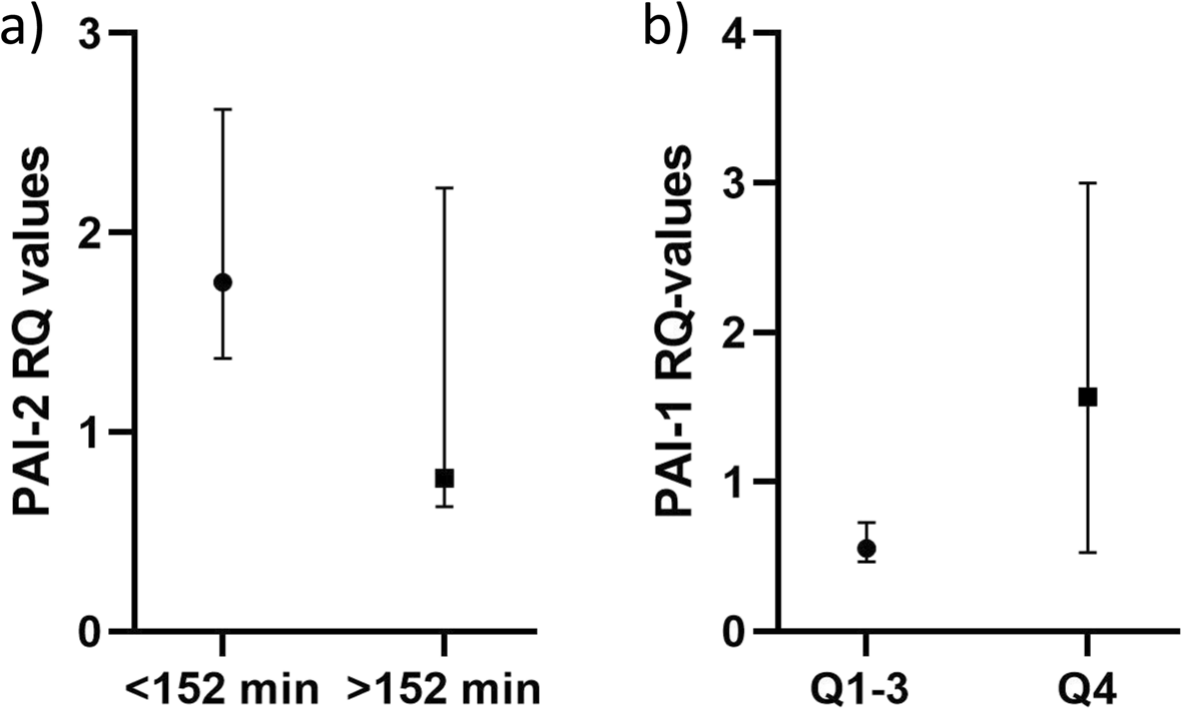
Relations to time from symptom to PCI. Panel **a** shows genes encoding PAI-2 in circulating leukocytes at time of PCI dichotomized at median time from symptom to PCI. Panel **b** shows genes encoding PAI-1 in circulating leukocytes at Day 1 dichotomized in Q1–3 vs Q4

As shown in Table 3, there were no significant correlations between genes expressed in thrombi and time from symptom to PCI. Genes in circulating leukocytes and PAI-1 in circulation, at either timepoint, also showed no correlations with time from symptom to PCI. However, when dividing time from symptom to PCI at median (152 min), genes encoding PAI-2 in circulating leukocytes at time of PCI were 1.2-fold higher (*p* = 0.048) in those with the shortest time to PCI (Fig. 3 panel a). Comparing Q1–3 vs Q4 of time from symptom to PCI, expression of PAI-1 in circulating leukocytes at Day 1 was 2.8-fold higher in Q4 (*p* = 0.040) (Fig. 3 panel b).

**Table 3:** Relation to time from symptom to PCI

At time of PCI						
	Genes in thrombus		Genes in circ.leuk.		Circulating marker	
	Rho	*p*	Rho	*p*	Rho	*p*
tPA	0.139	0.507	−0.121	0.540	–	–
uPA	0.062	0.773	−0.237	0.185	–	–
PAI-1	0.237	0.208	0.126	0.484	−0.038	0.832
PAI-2	−0.015	0.947	−0.222	0.214	–	–
At Day 1						
	Genes in thrombus		Genes in circ.leuk.		Circulating marker	
	Rho	*p*	Rho	*p*	Rho	*p*
tPA			−0.245	0.227	–	–
uPA			0.005	0.979	–	–
PAI-1			0.266	0.164	−0.266	0.142
PAI-2			−0.071	0.719	–	–

Coefficients of correlation (Spearmans rho) between time from symptom to PCI and the markers measured at time of PCI and at Day 1. *p* ≤ 0.05 bolded as sign of statistical significance

#### Changes in levels of circulating markers and gene expression in circulating leukocytes from time of PCI to the next morning (Day 1)

**Fig. 4:**
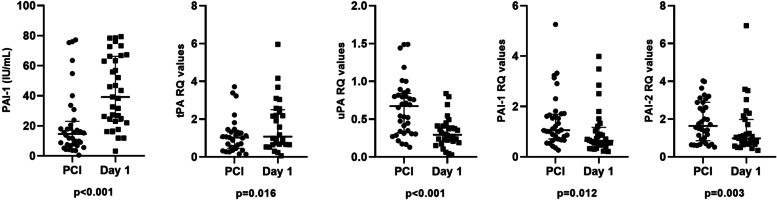
Changes from time of PCI to Day 1. The first graph shows changes from time of PCI to Day 1 in circulating PAI-1. The next four graphs show changes in genes expressed in circulating leukocytes from time of PCI to Day 1 for tPA, uPA, PAI-1 and PAI-2 (Wilcoxon rank sum test). Dots represent individual data. Horizontal lines are drawn at medians, and 25th and 75th percentiles

As shown in Fig. 4 (and Supplementary Table 5, Additional File 5), circulating PAI-1 was significantly higher the next day compared to time of PCI (*p* < 0.001). Also genetic expression of tPA in circulating leukocytes was significantly higher on Day 1 (*p* = 0.016), whereas mRNA levels of uPA, PAI-1 and PAI-2 in circulating leukocytes were all significantly lower on the next day (*p* < 0.001, *p* = 0.012 and *p* = 0.003, respectively). The changes (delta values) from time of PCI to Day 1 in circulating PAI-1 and genes in circulating leukocytes showed no associations with peak troponin T or time from symptom to PCI (data not shown).

#### Associations with cell markers (Table 4)

**Fig. 5:**
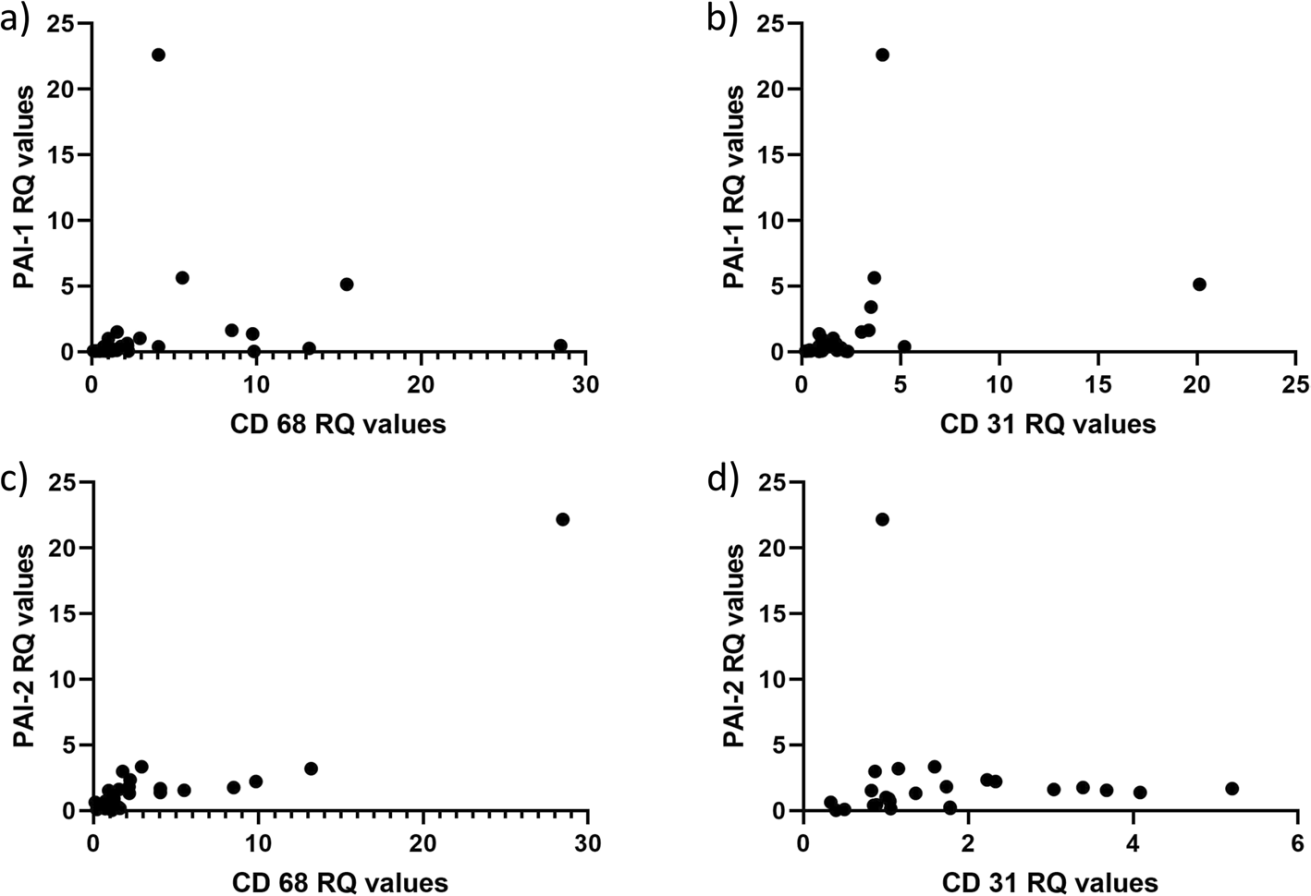
PAI-1 and PAI-2 in thrombi related to CD68 and CD31. Correlation between RQ-values of PAI-1 (Panel **a** and **b**) and PAI-2 (Panel **c** and **d**) in thrombi and RQ-values of CD 68 and CD 31

The expression of tPA, uPA, PAI-1 and PAI-2 in thrombi all showed significant correlations to CD68 (*p* = 0.028, *p* < 0.001, *p* = 0.003 and *p* < 0.001, respectively). Genes encoding PAI-1 and PAI-2 also correlated significantly to CD31 (*p* = 0.002 and *p* = 0.016, respectively), whereas expression of uPA correlated significantly with CD 66b (*p* = 0.019). However, number of thrombi with CD66b present was low (*n* = 6), and the relevance of this finding must therefore be considered uncertain. Figure 5 shows relationships between expression of PAI-1 (panel a and b) and PAI-2 (panel c and d), and CD 68 and CD 31 in thrombi. Recalculations after removal of outliers identified by inspection of plot were done, and had only limited effect on *p*-values and rho-values. No significant correlations between any of the fibrinolysis-related genes and CD3 or ACTA2/α-SMA were found.

**Table 4:** Genes in thrombi related to cell markers

	CD3		CD68		CD31		ACTA2		CD66b	
	Rho	*P*	Rho	*p*	Rho	*p*	Rho	*P*	Rho	*p*
**tPA**	−0.434	0.159	0.480	**0.028**	0.396	0.068	0.035	0.914	−0.029	0.957
**uPA**	0.073	0.805	0.757	**< 0.001**	0.358	0.086	0.242	0.426	0.886	**0.019**
**PAI-1**	0.213	0.464	0.543	**0.003**	0.557	**0.002**	0.350	0.221	0.086	0.872
**PAI-2**	0.222	0.446	0.751	**< 0.001**	0.496	**0.016**	0.168	0.602	0.314	0.544

Coefficients of correlations (Spearmans rho) between genetic expression of the Fibrinolytic markers and markers identifying different cell types in coronary thrombi. CD 3 = T-cell-marker. CD68 = Monocyte/Macrophage marker. CD31 = Endothelial cell marker. ACTA2/α-SMA = Smooth muscle cell marker. CD66b = Neutrophil granulocyte marke

#### Associations with diabetes type II

Levels of circulating PAI-1 at time of PCI were 4.0-fold higher in the group of patients with type 2 diabetes (*n* = 4) vs without (*n* = 29) (*p* = 0.018) (Supplementary Fig. 1, Additional File 8). At Day 1, levels of PAI-1 were still higher in patients with diabetes, but not statistically significant (*p* = 0.393). There were no differences in gene expression levels in thrombi or in circulating leukocytes at either time point for patients with type 2 diabetes vs without.

### Histology and immunohistochemistry

Twenty-seven of the 33 aspirated thrombi contained sufficient material for morphological studies and immunohistochemical analyses.

#### Immunohistochemistry analyses

Immunohistochemistry staining of PAI-1 were found in 72% of thrombi, whereas tPA, uPA and PAI-2 were present in 100%, as shown in Fig. 1c).

**Fig. 6:**
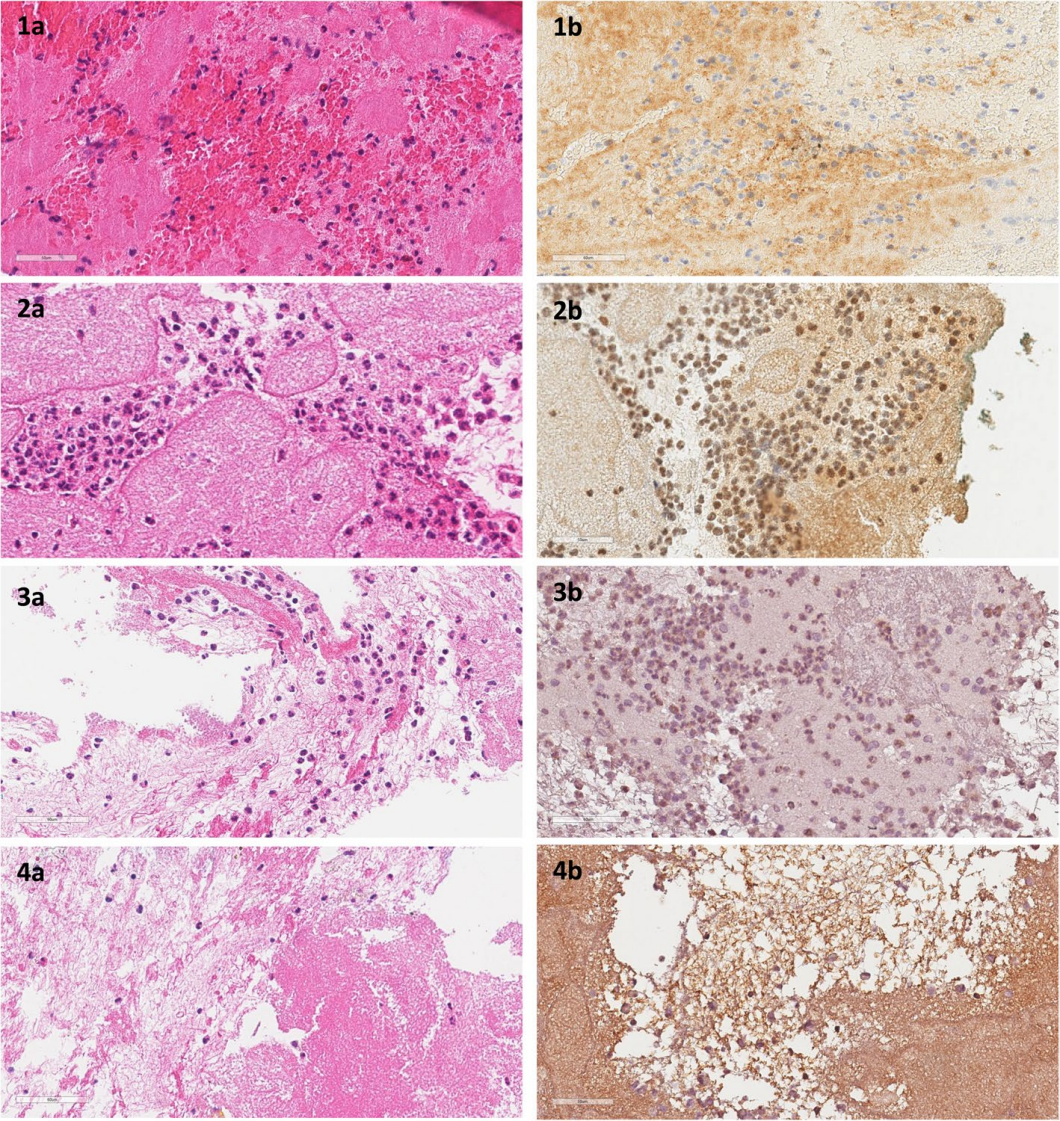
Histology and immunohistochemistry staining in thrombi

uPA, PAI-1 and PAI-2 were present in cytoplasm and at the membrane of monocytes and neutrophils, whereas tPA was found mainly in the cytoplasm of neutrophils and some monocytes. All markers were abundantly present extracellularly, especially in the fibrin surrounding the inflammatory cells (Supplementary Table 6, Additional File 6). Figure 6 shows typical immunohistochemistry staining of the four different markers and the corresponding HE-picture.

Histology and immunohistochemistry (brown signal) of thrombus. Initial magnification × 40, but pictures downsized afterwards. The panels show the following stainings: **1a** HE, erythrocytes, fibrin, intact- and partially karyorrhexitic granulocytes and monocytes. Stage 1 + 2. **1b** PAI-1 (brown signal), present in cytoplasm and extracellular of neutrophiles and monocytes. Stage 1 + 2. **2a** HE erythrocytes, fibrin, intact- and partially karyorrhexitic granulocytes and monocytes. Stage 1. **2b** PAI-2 (brown signal), present in cytoplasm and extracellular of neutrophiles and monocytes. Stage 1. **3a** HE, erythrocytes, fibrin, intact- and partially karyorrhexitic granulocytes and monocytes. Stage 1 + 2. **3b** uPA (brown signals), present in cytoplasm and extracellular of neutrophiles and monocytes. Stage 1 + 2. **4a** HE, erythrocytes, fibrin, intact- and partially karyorrhexitic granulocytes. Stage 1+. **4b** tPA (brown signals), present in cytoplasm and extracellular of neutrophiles and some monocytes. Stage 1 + .

#### Thrombus age

The histological staging into different age groups has been described previously [16]. Five (18.5%) of the thrombi were categorized as stage 1 (< 1 day old), six (22.2%) as stage 2 (1–5 days old) and two (7.4%) as stage 1+ (1 day + old). Fourteen (51.9%) showed histological characteristics of both stage 1 and stage 2, and were classified as stage 1 + 2. This indicates a process of repeated episodes with fresh bleeding and new thrombus expansion within a lytic thrombus. None of the retrieved thrombi were morphologically organized (Stage 3) according to the classification scheme (Supplementary Table 1, Additional File 1). The histological newest thrombi were associated with higher levels of peak troponin T than histological older thrombi, but not statistically significant (*p* = 0.068 comparing stage 1 vs stage 2). No differences were found for ischemic time between the different histological age groupings (data not shown).

Comparing levels of genes in thrombi between the different histological stages of thrombus age (Supplementary Table 7, Additional File 7), we found that tPA was higher expressed in histological older thrombi, statistically significant when comparing thrombi stage 2 vs the rest of thrombi (*p* = 0.041) and stage 1 vs stage 2 (*p* = 0.043), and non-significant comparing stage 1 vs the rest (*p* = 0.106). No coherent differences for the other genes in thrombi were found, or for any of the gene levels in circulating leukocytes. For circulating PAI-1, we found significantly higher levels at time of PCI in the patients with the morphologically newest thrombi when comparing stage 1 vs stage 2 (*p* = 0.018). When comparing stage 1 vs all other stages the difference was close to significant (*p* = 0.053), but not when comparing stage 2 vs other stages (*p* = 0.448).

## Discussion

The main finding in our study was that the fibrinolytic markers tPA, uPA, PAI-1 and PAI-2 are present and genetically upregulated in coronary thrombi from STEMI patients, and also expressed in circulating leukocytes. None were related to troponin T or to ischemic time. All markers were associated with the presence of monocytes/macrophages in thrombi.

Although genes of the investigated markers were highly expressed both in the thrombi and in circulating leukocytes at the time of PCI, we found no significant associations between the expression levels of the same genes in thrombi and circulating leukocytes. Thus the expression in the circulating cells seems not to reflect what is ongoing in the thrombi. This is also supported by the lack of correlations to circulating PAI-1, the only marker we measured in circulation.

There was a discrepancy between immunohistochemistry staining and genetic expression of the markers in the thrombi, especially regarding PAI-1, which was found upregulated in nearly 97%, but immunohistochemically stained in only 72%. This can be due to the fact that histopathological analyses only look at parts or slices of the thrombus, whereas genetic analyses represent the whole thrombus-part which from RNA was isolated. For the other markers, the relation was in contrast, higher percentage of thrombi with positive immunohistochemistry staining than with genetic upregulation. This could imply that not all proteins found in thrombi by immunohistochemistry are produced locally, but can also be due to methodological issues.

We did not find any significant associations between expression of the investigated markers in thrombi and circulating leukocytes towards degree of myocardial injury measured by peak troponin T. This was also the case for circulating PAI-1. Correlation between circulating PAI-1 and myocardial injury assessed by troponin T in patients with STEMI was shown in a larger study than the present study [12], but the correlation observed was weak. Thus, it might be that any association and importance of the specific fibrinolytic components with regard to myocardial damage is weak, and that our population is too small and selected.

Furthermore, we did not find consistent associations between the expression levels of the investigated markers in thrombi and leukocytes and time from symptom to PCI, as was also observed for circulating PAI-1. Only a tendency for decreased levels along with ischemic time was observed for genes encoding PAI-2 in circulating leukocytes at time of PCI, and 1.8-fold higher levels of PAI-1 were found in the highest quartile of ischemic time in circulating leukocytes at Day 1. This is opposed to a previous report of coronary thrombi showing tPA, uPA and PAI-1 expression to be associated with ischemic time [21]. In that study however, ischemic time was much longer, with a median of 240 min, vs 152 min in our study, making any potential relation to time more likely to be detected. Longer ischemic time will also give more time for progression of thrombosis and fibrinolysis, and also upregulation of genetic expression of the fibrinolytic markers to take place locally. Our findings of different histopathological age-stages within the same thrombi in more than half of the cases suggests that intracoronary thrombi can develop stepwise, and that thrombus formation probably often is a process exceeding over time. It should also be emphasized that time from symptom onset to PCI is an inaccurate clinical parameter, as many patients have on-and-off symptoms, which makes the data uncertain. We did however, find higher expression of tPA in histologically older thrombi, indicating upregulation of the fibrinolytic system along with time in myocardial infarction.

We found that levels of mRNA in circulating leukocytes encoding uPA, PAI-1 and PAI-2 were lower the next day when compared to time of PCI, whereas circulating PAI-1 was higher. This is in line with previous reports, studying gene regulation of inflammatory markers [16] and metalloproteinases [22] in coronary thrombi, and could indicate negative-feedback mechanisms in the leukocytes. However, genes encoding tPA were higher on Day 1. This may be an upregulation due to a rapid binding of tPA to PAI-1 as well as to fibrin in the thrombi, and thus a feed-back mechanism in the leukocytes. This can also be seen in line with the increase in circulating PAI-1 levels from time of PCI to Day 1, with a compensatory upregulation of tPA. As PAI-1 is an acute phase protein, a rapid increase is expected. The increase in PAI-1 was however not accompanied by increased leukocyte gene expression, and we also even found an inverse association between genes encoding PAI-1 in circulating leukocytes at time of PCI and level of circulating PAI-1 at Day 1. This can be explained by the fact that PAI-1 is produced by several cell types and tissues, including endothelial cells, platelets, adipose tissue and liver [23], which probably contributes substantially more to circulating levels than leukocytes.

The expression in thrombi of all investigated markers showed close association to markers of inflammatory cells, especially monocytes, and for u-PA also neutrophil granulocytes, although these were present to a limited degree. This was in conjunction with our histological findings of the markers being localized largely in proximity of inflammatory cells. Taken together with the observation of no significant associations between the expression levels of the same genes in thrombi and circulating leukocytes, this indicates in general a close connection of the fibrinolytic system and local inflammatory cells. This supports existing theories of interplay between the immune system and the fibrinolytic system, especially u-PA, regulating leukocyte extravasation and recruitment to sites of inflammation [24]. There is also evidence of tPA recruiting leukocytes in ischemia-reperfusion (I/R) injury [25], mediated both by direct proteolytic and non-proteolytic abilities, and also indirectly through activation of gelatinases by activated plasmin. We found correlations between genetic expression of PAI-1 and PAI-2, and the endothelial cell marker CD31, which matches well with previous findings of PAI-1 accumulating on endothelial cells and mediating pro-inflammatory responses in the setting of I/R-injury [26]. This implies that PAI-1 and PAI-2 in thrombi probably are produced by locally situated inflammatory cells in proximity to endothelial cells, by endothelial cells directly, or, most likely, both. Somewhat surprisingly, tPA was not associated with CD31, as endothelial cells under normal conditions are the main source of tPA.

Although low number, we found higher levels of circulating PAI-1 in patients with diabetes type 2 vs without. This is in line with previous evidence [9], supporting our results.

### Limitations

A major limitation is the small sample size of our study, making adjustments for known risk factors and studying sub-groups difficult. Only 3 (9%) were female, not representative for a typical cohort of patients with STEMI. Patients included in the TASTI study are a selection of STEMI patients, considered to profit from thrombus aspiration as evaluated by the PCI-operator in charge of the procedure. Hence, patients included represent a subgroup with an extra tendency for thrombus formation, and the generalizability of the study results can be discussed. Only PAI-1 was measured in the circulation. Measure of tPA activity, which could have added to the results, is challenging as special immediately handling of the blood sample is needed. Measure of the amount tPA as tPA-antigen could be performed, however, as this mainly represents PAI-1/tPA-complex, it would not add relevant information. Although isolated RNA from the thrombi showed satisfactory purity and amount as measured by Nanodrop, lack of RNA quality and integrity measures, is a limitation.

Nearly all patients were given intravenous heparin. Heparins main effects are on the coagulation cascades, mediated through inhibition of factor Xa and thrombin, but it has also been reported heparin-associated reduction in PAI-1 levels in patients with acute coronary syndrome (ACS) patients [27], as well as stimulation of tPA-release form endothelial cells by heparin [28]. All patients were also given platelet-inhibition with acetylsalicylic acid and clopidogrel orally before arriving at the hospital, as well as gpIIb/IIIa antagonist during the procedure, before the thrombi were aspirated. This may have influenced the results, as there previously has been reported on active platelets producing large amounts of active PAI-1 [29]. The markers analyzed are a selection based on existing knowledge in the field, but from a broad range of mediators potentially involved in responses in myocardial ischemic disease. Peak troponin T as the only indicator of myocardial injury is also a limitation. A strength in our study is the combination of studying both genetic expression and actual presence of the investigated markers in human material.

## Conclusions

Genes encoding tPA, uPA, PAI-1 and PAI-2 were highly expressed in human coronary thrombi from STEMI patients, indicating fibrinolytic regulators to play active roles in the thrombi, although not related to myocardial injury. All markers associated especially to the presence of monocytes/macrophages, indicating these markers to be connected to local inflammatory cells.

## Supplementary Information

**Additional file 1: Supplementary Table 1.** Displays characteristics of the different histological age stages used for classifying thrombi into different age stages.

**Additional file 2: Supplementary Table 2.** Listing of the antibodies used for immunohistochemistry analyzes.

**Additional file 3: Supplementary Table 3.** Shows gene expression levels in the aspirated thrombi, circulating leukocytes and serum levels.

**Additional file 4: Supplementary Table 4.** Displays correlations between circulating PAI-1 and corresponding genes expressed in thrombi and in circulating leukocytes.

**Additional file 5: Supplementary Table 5.** Shows changes in levels of circulating PAI-1 and gene expression in circulating leukocytes from time of PCI to Day 1.

**Additional file 6: Supplementary Table 6.** Displays localization of the fibrinolytic markers by immunohistochemistry staining.

**Additional file 7: Supplementary Table 7.** Shows data grouped according to histological age stages, with comparison of groups.

**Additional file 8: Supplementary Figure 1.** Shows levels of circulating PAI-1 at time of PCI grouped in DM vs non-DM.

## Data Availability

The datasets analysed during the current study are available from the corresponding author on reasonable request.

## Abbreviations

ACS: Acute Coronary Syndrome
cTnT: Cardiac troponin T
HE: Hematoxylin-eosin
I/R: Ischemia-reperfusion
LAD: Left anterior descending artery
MI: Myocardial infarction
PAI-1: Plasminogen-activator inhibitor type 1
PAI-2: Plasminogen-activator inhibitor type 2
PCI: Percutaneous coronary intervention
Q: Quartile
RQ: Relative quantification
STEMI: ST-elevation myocardial Infarction
tPA: Tissue plasminogen activator
uPA: Urinary-type plasminogen activator
