# Supplementary material for: Gene expression of fibrinolytic markers in coronary thrombi
Source: Thromb J. 2022 Apr 29;20:23. doi: 10.1186/s12959-022-00383-1 (PMC9052700; doi:10.1186/s12959-022-00383-1)
Supplement: Supplementary file 1 — Additional file 1: Supplementary Table 1. Displays characteristics of the different histological age stages used for classifying thrombi into different age stages. [file 12959_2022_383_MOESM1_ESM.docx]

***Supplementary Table 1. Histologic thrombus age stages.***

| Thrombus stage |  | Estimated age | Histologic characteristics |
| --- | --- | --- | --- |
| Stage 1 | Fresh | < 1 day | Platelet aggregates, erythrocytes/fresh bleeding, intact granulocytes in focal areas, fibrin of varying organization |
| Stage 1+ |  | 1 day + | Platelet aggregates, erythrocytes/fresh bleeding, intact granulocytes in focal areas and fibrin of varying organization, small areas with disintegration of granulocytes |
| Stage 2 | Lytic | 1-5 days | Areas of colliquative (liquefactive) necrosis and diffuse spreading of granulocyte with karyorrhexis and necrosis, increasing number of monocytes. |
| Stage 3 | Organized | > 5 days | Presence of smooth muscle cells, homogeneous or hyaline fibrin. Depositions of fibroblasts and endothelial cells. |

Estimated age and histologic characteristics of the different thrombus age stages.
