# Supplementary material for: Gene expression of fibrinolytic markers in coronary thrombi
Source: Thromb J. 2022 Apr 29;20:23. doi: 10.1186/s12959-022-00383-1 (PMC9052700; doi:10.1186/s12959-022-00383-1)
Supplement: Supplementary file 2 — Additional file 2: Supplementary Table 2. Listing of the antibodies used for immunohistochemistry analyzes. [file 12959_2022_383_MOESM2_ESM.docx]

***Supplementary Table 2. Antibodies.***

| **Antibody** | **Source** | **Dilution** |
| --- | --- | --- |
| tPA | Abcam, ab 47742 | 1/1000 |
| uPA | Abcam, ab 66705 | 1/500 |
| PAI-1 | Abcam, ab 1574 | 1/500 |
| PAI-2 | R&D, AF 1310 | 1/100 |

Listing of antibodies used for immunohistochemistry analyzes.
