# Supplementary material for: Gene expression of fibrinolytic markers in coronary thrombi
Source: Thromb J. 2022 Apr 29;20:23. doi: 10.1186/s12959-022-00383-1 (PMC9052700; doi:10.1186/s12959-022-00383-1)
Supplement: Supplementary file 3 — Additional file 3: Supplementary Table 3. Shows gene expression levels in the aspirated thrombi, circulating leukocytes and serum levels. [file 12959_2022_383_MOESM3_ESM.docx]

***Supplementary Table 3. Gene expression levels.***

|  | RQ values  Thrombi | RQ values  Leukocytes  At PCI | RQ values  Leukocytes  Day1 | Circulating levels (IU/mL)  At PCI | Circulating levels (IU/mL)  Day 1 |
| --- | --- | --- | --- | --- | --- |
| tPA | 0.11 (0.03, 0.41) | 1.02 (0.48, 1.29) | 1.38 (0.65, 2.55) | - | - |
| uPA | 5.61 (1.54, 15.2) | 0.65 (0.34, 0.85) | 0.30 (0.21, 0.41) | - | - |
| PAI-1 | 0.38 (0.07, 1.02) | 1.05 (0.71, 1.62) | 0.56 (0.47, 1.22) | 15.3 (7.54, 20.5) | 35.4 (22.5, 63.1) |
| PAI-2 | 1.33 (0.41, 1.76) | 1.51 (0.83, 2.56) | 0.85 (0.67, 1.22) | - | - |

Gene expression levels (RQ) in the aspirated thrombi, circulating leukocytes and serum levels. Medians (25, 75 percentiles) are given.
