# Supplementary material for: Gene expression of fibrinolytic markers in coronary thrombi
Source: Thromb J. 2022 Apr 29;20:23. doi: 10.1186/s12959-022-00383-1 (PMC9052700; doi:10.1186/s12959-022-00383-1)
Supplement: Supplementary file 4 — Additional file 4: Supplementary Table 4. Displays correlations between circulating PAI-1 and corresponding genes expressed in thrombi and in circulating leukocytes. [file 12959_2022_383_MOESM4_ESM.docx]

***Supplementary Table 4. Circulating markers towards genes.***

|  | **GENES IN THROMBUS** | |  |  | **GENES IN CIRC LEUK** | | |
| --- | --- | --- | --- | --- | --- | --- | --- |
| Circulation |  |  |  | At PCI |  | Day 1 |  |
|  | Rho | p |  | Rho | P | Rho | p |
| PAI-1 at PCI | -0.004 | 0.982 |  | 0.181 | 0.314 | -0.005 | 0.980 |
| PAI-1 Day 1 | -0.106 | 0.583 |  | -0.399 | **0.024** | -0.261 | 0.171 |

Correlations between circulating PAI-1 and corresponding genes expressed in thrombi and in circulating leukocytes (Spearmans rho). p≤0.05 bolded as sign of statistical significance.
