# Supplementary material for: Gene expression of fibrinolytic markers in coronary thrombi
Source: Thromb J. 2022 Apr 29;20:23. doi: 10.1186/s12959-022-00383-1 (PMC9052700; doi:10.1186/s12959-022-00383-1)
Supplement: Supplementary file 5 — Additional file 5: Supplementary Table 5. Shows changes in levels of circulating PAI-1 and gene expression in circulating leukocytes from time of PCI to Day 1. [file 12959_2022_383_MOESM5_ESM.docx]

***Supplementary Table 5. Changes from time of PCI to Day 1.***

|  |  |  | At PCI | Day 1 | p |
| --- | --- | --- | --- | --- | --- |
| CIRCULATING MARKERS (IU/mL) | |  |  |  |  |
| PAI-1 |  |  | 15.3 (7.54,20.5) | 35.4 (22.5, 63.1) | **<0.001** |
| GENES IN CIRC LEUK (RQ values) | |  |  |  |  |
| tPA |  |  | 1.02 (0.48, 1.29) | 1.38 (0.65, 2.55) | **0.016** |
| uPA |  |  | 0.65 (0.34, 0.85) | 0.30 (0.21, 0.41) | **<0.001** |
| PAI-1 |  |  | 1.05 (0.71, 1.62) | 0.56 (0.47, 1.22) | **0.012** |
| PAI-2 |  |  | 1.51 (0.83, 2.56) | 0.85 (0.67, 1.22) | **0.003** |

Levels of circulating markers and gene expression in circulating leukocytes at time of PCI and at Day 1 (Wilcoxon Rank sum test). Data are given as median (25^th^, 75^th^ percentile). p≤0.05 bolded as sign of statistical significance.
