# Supplementary material for: Gene expression of fibrinolytic markers in coronary thrombi
Source: Thromb J. 2022 Apr 29;20:23. doi: 10.1186/s12959-022-00383-1 (PMC9052700; doi:10.1186/s12959-022-00383-1)
Supplement: Supplementary file 6 — Additional file 6: Supplementary Table 6. Displays localization of the fibrinolytic markers by immunohistochemistry staining. [file 12959_2022_383_MOESM6_ESM.docx]

***Supplementary Table 6. Localization by immunohistochemistry staining.***

| **Immune marker** | **Cell type** | **Localization** |
| --- | --- | --- |
| uPA | Monocytes and neutrophils | Cytoplasm, membrane and extracellularly. |
| tPA | Neutrophils and some monocytes | Cytoplasm and extracellularly |
| PAI-1 | Monocytes and neutrophils | Cytoplasm, membrane and extracellularly. |
| PAI-2 | Monocytes and neutrophils | Cytoplasm, membrane and extracellularly. |

Cell types, and localization within the cells, of the different markers visualized with immunohistochemistry staining.
