# Supplementary material for: Gene expression of fibrinolytic markers in coronary thrombi
Source: Thromb J. 2022 Apr 29;20:23. doi: 10.1186/s12959-022-00383-1 (PMC9052700; doi:10.1186/s12959-022-00383-1)
Supplement: Supplementary file 7 — Additional file 7: Supplementary Table 7. Shows data grouped according to histological age stages, with comparison of groups. [file 12959_2022_383_MOESM7_ESM.docx]

***Supplementary Table 7. Differences between histologic age stages.***

Data grouped according to histologic age stages. a: 1 vs other; b: 2 vs other; c: 1 vs 2. Groups compared by Mann Whitney U test. Data are givens as medians (25^th^, 75^th^-percentiles).

a)

|  |  | At PCI |  |  | Day 1 |  |  |
| --- | --- | --- | --- | --- | --- | --- | --- |
|  |  | Stage 1 | Other | p | Stage 1 | Other | p |
| **Genes in thrombus** | |  |  |  |  |  |  |
| tPA |  | 0.04 (0.01, 0.08) | 0.12 (0.03, 0.59) | 0.106 |  |  |  |
| uPA |  | 15.4 (1.58, 29.3) | 6.55 (1.50, 20.1) | 0.905 |  |  |  |
| PAI-1 |  | 0.07 (0.04, 0.20) | 0.46 (0.11, 1.36) | **0.045** |  |  |  |
| PAI-2 |  | 0.64 (0.16, 2.22) | 1.39 (0.46, 1.67) | 0.634 |  |  |  |
| **Markers in circulation** | |  |  |  |  |  |  |
| PAI-1 |  | 18.4 (17.9, 33.7) | 9.47 (6.77, 18.0) | 0.053 | 56.9 (39.2, 63.0) | 26.9 (17.6, 55.9) | 0.143 |
| **Genes in leukocytes** | |  |  |  |  |  |  |
| tPA |  | 0.82 (0.50, 1.04) | 1.07 (0.36, 1.32) | 0.417 | 2.35 (1.49, 2.56) | 1.28 (0.59, 3.07) | 0.637 |
| uPA |  | 0.71 (0.48, 0.85) | 0.54 (0.34, 0.80) | 0.662 | 0.36 (0.19, 0.38) | 0.29 (0.21, 0.41) | 0.941 |
| PAI-1 |  | 1.00 (0.72, 1.21) | 1.06 (0.67, 1.72) | 0.574 | 0.36 (0.23, 0.48) | 0.68 (0.56, 1.51) | 0.053 |
| PAI-2 |  | 2.44 (1.86, 2.89) | 1.40 (0.70, 2.01) | 0.092 | 0.67 (0.64, 1.12) | 0.85 (0.79, 1.24) | 0.724 |

Thrombustsage 1, n = 5. Thrombusstage Other (1+, 1+2, 2), n = 22. p≤0.05 bolded as sign of statistical significance.

b)

|  |  | At PCI |  |  | Day 1 |  |  |
| --- | --- | --- | --- | --- | --- | --- | --- |
|  |  | Stage 2 | Other | p | Stage 2 | Other | p |
| **Genes in thrombus** | |  |  |  |  |  |  |
| tPA |  | 0.63 (0.35, 6.12) | 0.06 (0.02, 0.15) | **0.041** |  |  |  |
| uPA |  | 6.55 (4.59, 20.1) | 7.16 (1.54, 19.8) | 0.934 |  |  |  |
| PAI-1 |  | 1.36 (0.24, 1.64) | 0.35 (0.07, 0.82) | 0.308 |  |  |  |
| PAI-2 |  | 1.46 (0.92, 1.65) | 1.17 (0.34, 1.74) | 0.777 |  |  |  |
| **Markers in circulation** | |  |  |  |  |  |  |
| PAI-1 |  | 10.5 (8.00, 15.7) | 15.5 (6.85, 23.9) | 0.448 | 24.9 (16.5, 43.5) | 38.3 (24.5, 63.3) | 0.283 |
| **Genes in leukocytes** | |  |  |  |  |  |  |
| tPA |  | 0.81 (0.28, 1.32) | 1.03 (0.64, 1.15) | 0.834 | 3.04 (0.30, 4.16) | 1.58 (0.83, 2.55) | 0.491 |
| uPA |  | 0.52 (0.31, 0.77) | 0.54 (0.42, 0.82) | 0.560 | 0.35 (0.27, 0.39) | 0.29 (0.17, 0.45) | 0.685 |
| PAI-1 |  | 1.09 (1.05, 1.82) | 1.01 (0.67, 1.62) | 0.414 | 1.06 (0.58, 1.66) | 0.56 (0.36, 1.22) | 0.465 |
| PAI-2 |  | 1.10 (0.70, 2.56) | 1.57 (1.00, 2.44) | 0.521 | 0.91 (0.82, 2.00) | 0.82 (0.57, 1.24) | 0.444 |

Thrombusstage 2, n = 6. Thrombusstage Other (1, 1+, 1+2), n = 21

c)

|  |  | At PCI |  |  | Day 1 |  |  |
| --- | --- | --- | --- | --- | --- | --- | --- |
|  |  | Stage 1 | Stage 2 | p | Stage 1 | Stage 2 | p |
| **Genes in thrombus** | |  |  |  |  |  |  |
| tPA |  | 0.04 (0.01, 0.08) | 0.63 (0.35, 6.12) | **0.043** |  |  |  |
| uPA |  | 15.4 (1.58, 29.3) | 6.55 (4.59, 20.1) | 1.000 |  |  |  |
| PAI-1 |  | 0.07 (0.04, 0.20) | 1.36 (0.24, 1.64) | 0.142 |  |  |  |
| PAI-2 |  | 0.64 (0.16, 2.22) | 1.46 (0.92, 1.65) | 0.724 |  |  |  |
| **Markers in circulation** | |  |  |  |  |  |  |
| PAI-1 |  | 18.4 (17.9, 33.7) | 10.5 (8.00, 15.7) | **0.018** | 56.9 (39.2, 63.0) | 24.9 (16.5, 43.5) | 0.076 |
| **Genes in leukocytes** | |  |  |  |  |  |  |
| tPA |  | 0.82 (0.50, 1.04) | 0.84 (0.28, 1.32) | 0.831 | 2.36 (1.49, 2.56) | 3.04 (0.30, 4.16) | 0.480 |
| uPA |  | 0.71 (0.48, 0.85) | 0.52 (0.31, 0.77) | 0.584 | 0.36 (0.19, 0.38) | 0.35 (0.27, 0.39) | 0.907 |
| PAI-1 |  | 1.00 (0.72, 1.21) | 1.01 (1.05, 1.81) | 0.465 | 0.36 (0.23, 0.48) | 1.06 (0.58, 1.66) | 0.142 |
| PAI-2 |  | 2.44 (1.86, 2.89) | 1.11 (0.70, 2.56) | 0.144 | 0.67 (0.64, 1.12) | 0.91 (0.82, 2.00) | 0.327 |

Thrombustage 1, n = 5. Thrombustage 2, n = 6. p≤0.05 bolded as sign of statistical significance.
