# Supplementary figures and images for: Gene expression of fibrinolytic markers in coronary thrombi
Source: Thromb J. 2022 Apr 29;20:23. doi: 10.1186/s12959-022-00383-1 (PMC9052700; doi:10.1186/s12959-022-00383-1)

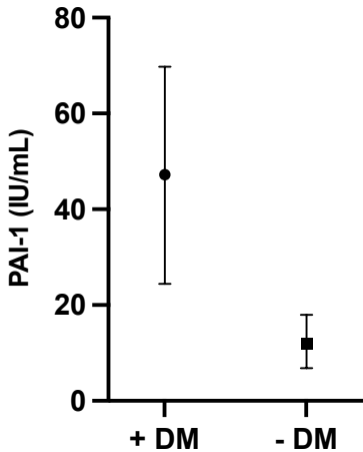

Supplement: Supplementary file 8 — Additional file 8: Supplementary Figure 1. Shows levels of circulating PAI-1 at time of PCI grouped in DM vs non-DM. [file 12959_2022_383_MOESM8_ESM.pdf]
